# Acute Hemorrhagic Leukoencephalitis in a Patient With Hepatitis B

**DOI:** 10.7759/cureus.67587

**Published:** 2024-08-23

**Authors:** Dhiran Sivasubramanian, Mohamed Raghib Hussain Mohamed Kalifa

**Affiliations:** 1 Critical Care Medicine, Christian Medical College, Vellore, IND; 2 Cardiology, MGM Healthcare, Chennai, IND

**Keywords:** seronegative autoimmune encephalitis, autoimmune encephalitis, post-viral encephalitis, mri images, multiple sclerosis flare treatment, tumefactive demyelination, acute disseminated encephalomyelitis (adem), hepatitis b infection, acute hemorrhagic leukoencephalitis, weston-hurst syndrome

## Abstract

Acute hemorrhagic leukoencephalitis (AHLE), also known as Weston-Hurst syndrome or Hurst disease, is a rare and rapidly progressive form of acute disseminated encephalomyelitis. It is characterized by severe inflammation, hemorrhage, and necrosis within the white matter of the brain. AHLE often follows an upper respiratory infection or other systemic illnesses, suggesting a potential post-infectious autoimmune mechanism. The disease is associated with a high mortality rate and significant disability among survivors.

We present the case of a 46-year-old Indian woman with a history of chronic hepatitis B (HBV) who presented with an insidious onset of right-sided limb weakness and bi-frontal headaches. Initial brain MRIs showed features of tumefactive demyelination. Despite aggressive treatment with intravenous (IV) methylprednisolone, IV immunoglobulin, and anti-edema measures, the patient’s condition rapidly deteriorated, leading to a diagnosis of AHLE following the emergence of hemorrhagic white matter lesions on repeat MRI. Remarkably, with continued treatment, the patient survived and showed gradual neurological improvement, although she remained significantly debilitated at the time of discharge.

AHLE represents one of the most severe forms of demyelinating diseases, often resulting in rapid neurological decline and high mortality. This case highlights the potential link between chronic HBV infection with a high viral load and the onset of AHLE. The patient's recovery underscores the importance of early recognition and aggressive treatment in improving outcomes, even in conditions with traditionally poor prognosis.

Clinicians should maintain a high index of suspicion for AHLE in patients with chronic viral infections presenting with neurological symptoms. Prompt and aggressive management can be life-saving, and ongoing research is needed to better understand the pathogenesis and optimal treatment strategies for this rare but devastating condition.

## Introduction

Acute hemorrhagic leukoencephalitis (AHLE), also known as Weston-Hurst syndrome or Hurst disease, is a rare and devastating form of acute disseminated encephalomyelitis (ADEM). It is characterized by rapid progression and acute inflammation of the brain's white matter [1]. Initially described by Hurst in 1941, AHLE is marked by perivascular demyelination, hemorrhage, and necrosis within the white matter [1]. Although its etiology remains uncertain, AHLE often follows an upper respiratory infection [2] or other systemic illnesses [3], indicating a potential post-infectious autoimmune mechanism [1]. The disease leads to severe neurological deficits, coma, and death within the first week, with an estimated mortality rate of 70% [1,3] and a significant disability in those who survive [3,4]. AHLE presents significant challenges in both diagnosis and treatment.

In this case report, we discuss the clinical journey of a 46-year-old Indian woman with chronic hepatitis B (HBV) who developed headaches and limb weakness, ultimately leading to an AHLE diagnosis. Despite the critical nature of the disease, the patient has survived and is gradually recovering. Her case highlights the aggressive nature of this typically fatal disease and emphasizes that prompt recognition and treatment can improve survival chances. By documenting this case, we aim to enhance the understanding of AHLE and contribute to the limited existing literature on this condition.

## Case presentation

**Figure 1:**
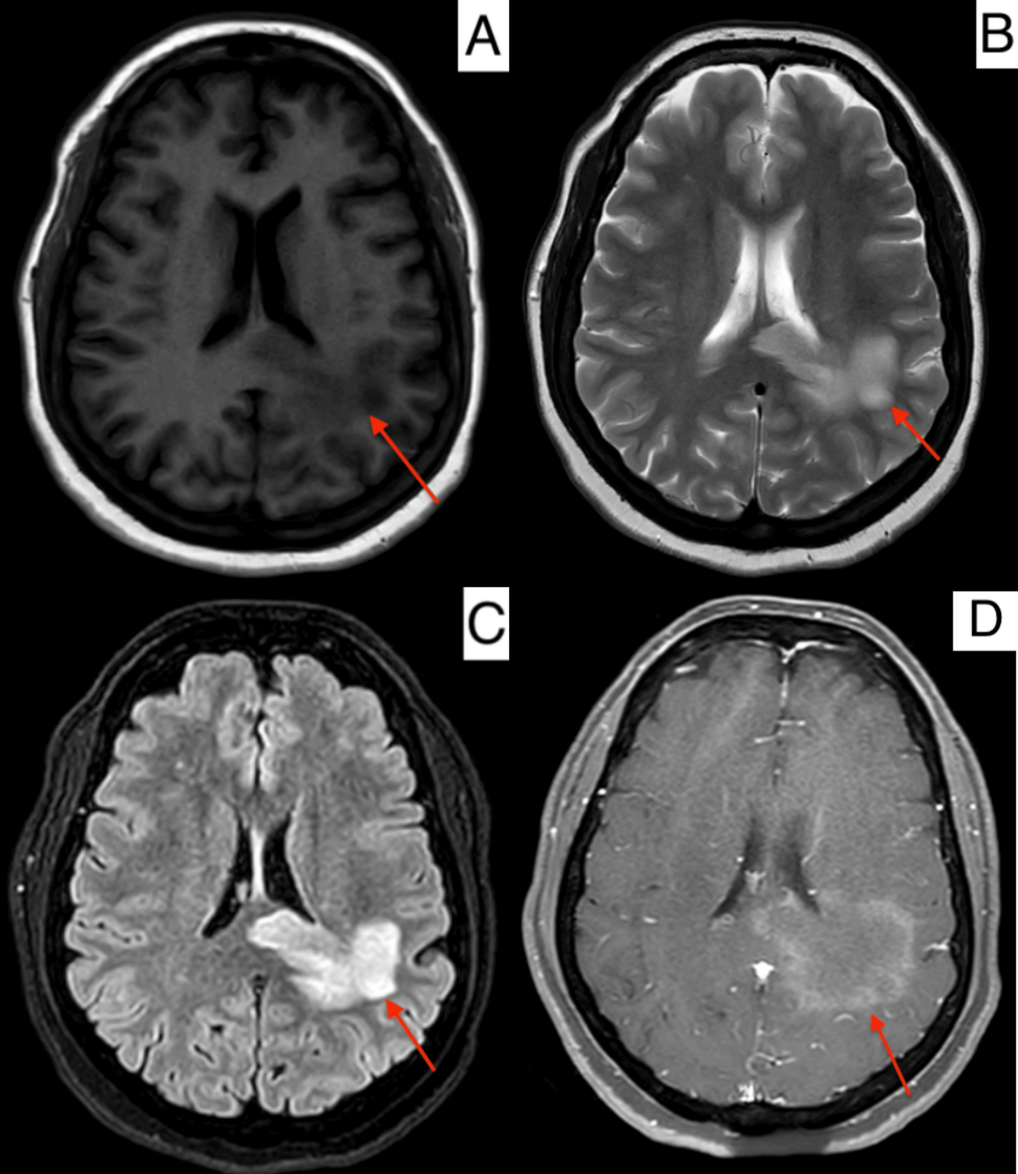
MRI of the brain. T1-weighted image (A), T2-weighted image (B), T2-FLAIR image (C), and T1-weighted contrast-enhanced image (D) showing tumefactive demyelination involving the left parietal region (red arrow) June 1, 2024 MRI: magnetic resonance imaging, FLAIR: fluid-attenuated inversion recovery

A 46-year-old female with a past medical history of type 2 diabetes mellitus, systemic hypertension, obstructive sleep apnea, chronic HBV, and coronary artery disease with prior percutaneous coronary intervention on dual antiplatelet therapy presented to the emergency department in the evening on June 6, 2024, with chief complaints of insidious onset of weakness in her right upper and lower limbs and bi-frontal headaches since early morning. She gives a history of intermittent headaches and persistent coughs for the past week, which were not associated with nausea, vomiting, or photophonophobia. She had a few episodes of giddiness lasting for a few minutes in the past week; there were no speech or visual disturbances. Her vitals on presentation were a blood pressure of 130/70 mmHg, a heart rate of 92 beats/min, a respiratory rate of 20/min, a temperature of 1010 F, and an oxygen saturation of 99%. On examination, she was conscious, alert, and oriented; the Glasgow coma scale was 15/15, and pupils were equally reactive to light and accommodation. There was right upper motor neuron type facial lag sparing the forehead muscles, power of 3/5 in the right upper limb and 1/5 in the right lower limb with brisk deep tendon reflexes 3+ on both limbs. All other cranial nerve functions were intact. The examination of the cardiopulmonary system and abdomen was normal. Routine blood investigation showed neutrophilic leukocytosis (Table 1) and an HbA1c of 9.3%, and all other values were within the reference range (Table 1). An MRI of the brain showed features suggestive of tumefactive demyelination involving the left parietal region (Figure 1). An MRI of the spine showed no abnormality. The patient was started on pulse steroid therapy with intravenous (IV) methylprednisolone [5], anti-seizure medications, and was continued on regular statin, but dual antiplatelet therapy was withheld and was started on IV heparin. Her glycemic levels were optimized adequately. Given the history of cough, a flu panel was sent, which came back negative. She was added to oral azithromycin and nebulizations.

**Table 1:** Routine hematological investigations

Type	Patient value	Reference value	Units
Hemoglobin	8.3	12-15	g/dL
Packed cell volume	27.6	40-50	%
Red blood cell count	3.51	3.8-5.8	x10^6/μL
Mean corpuscular volume (MCV)	78.6	81-9	fL
Mean corpuscular hemoglobin (MCH)	23.6	27-32	pg
Mean corpuscular hemoglobin concentration (MCHC)	30.1	31.5-34.5	%
Red blood cell distribution width (RDW)	17.1	11.5-14	%
White blood cell count	8.61	4-10	x10^3^/μL
Platelet count	197	150-400	x10^3^/μL
Differential count			
Neutrophils	88.6	40-80	%
Lymphocytes	7.1	20-40	%
Monocytes	4.2	2-10	%
Eosinophils	0.0	1-6	%
Basophils	0.1	0-2	%
Serum glucose random	240.3	80-140	mg/dL
Renal function test			
Creatinine	1.1	0.5-1.2	mg/dL
Urea	17	7-40	mg/dL
Electrolytes			
Sodium	131	136-145	mmol/L
Potassium	4.3	3.5-5.1	mmol/L
Chloride	97.9	98-111	mmol/L
Bicarbonate	22.4	22-29	mmol/L
Calcium	8.9	8.5- 10.5	mg/dL
Liver function test			
Total bilirubin	0.41	1.15	mg/dL
Direct bilirubin	0.23	0.01-0.7	mg/dL
Aspartate aminotransferase (AST)	16	5-42	U/L
Alanine aminotransferase (ALT)	11	5-42	U/L
Alkaline phosphatase (ALP)	77.3	35-129	U/L
Albumin	3.9	3.5-5.2	g/dL
Albumin/globulin (A/G) ratio	1.15	1.1-1.7	Ratio

The estimated sedimentation rate was normal; serum anti-nuclear antibody, antineutrophilic cytoplasmic antibody, anti-phospholipid antibody, anti-neuromyelitis optica antibody, and anti-myelin oligodendrocyte glycoprotein antibody were all negative. She had a persistent headache and was added to indomethacin, which led to moderate relief. A repeat MRI three days later showed a mild increase in the extent of left parietal white matter signal changes and complete resolution of the advancing margin. She was continued on IV pulse steroid therapy and limb physiotherapy. To rule out sarcoidosis [6], a CT chest was done, which showed no abnormality, and serum angiotensin-converting enzyme levels were within normal limits. In view of chronic HBV status, a viral load of 63,164 IU/mL was obtained, and liver function tests were within normal limits. The patient then underwent a lumbar puncture with cerebrospinal fluid (CSF) analysis, which showed normal cell count and protein, the results of which are seen in Table 2. To rule out progressive multifocal leukoencephalopathy, serum JC virus antibodies were sent, which came back negative. Serum and CSF oligoclonal banding were absent, ruling out multiple sclerosis (MS) [7]. A positron emission tomography CT was performed, which showed no evidence of any malignancy. CD4 T lymphocyte count via flow cytometry was normal.

**Table 2:** CSF analysis NA: not applicable, CSF: cerebrospinal fluid, IgG: immunoglobulin G

Type	Patient value	Reference value
Glucose	139.6 mg/dL	50-80
Protein	38.4 mg/dL	15-60
Red blood cell count	1000 cells/mm^3^	0-5
White blood cell count	2 cells/mm^3^	0-5
Neutrophils	50%	2-7
Lymphocytes	50%	16-56
Cell cytology	Negative	NA
CSF meningoencephalitis panel (Biofire)	Negative	NA
CSF human polyoma virus 2 (JC) virus	Negative	NA
CSF IgG index	0.65	0.25-0.7
CSF oligoclonal banding	Absent	NA

**Figure 2:**
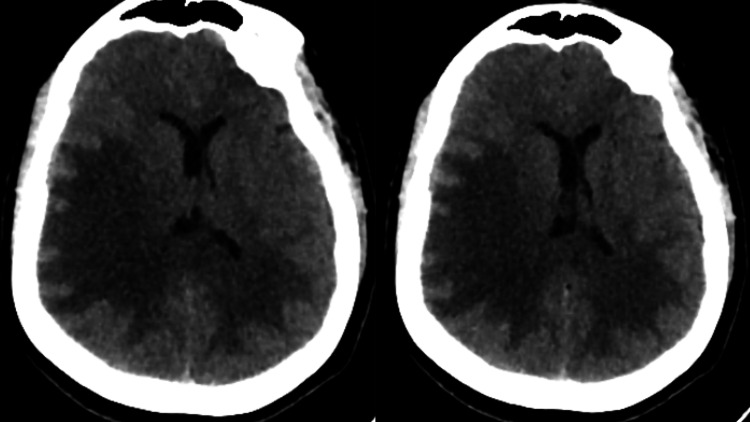
CT of the brain showing new white matter hypodensity involving the right cerebral hemisphere, causing a mass effect and midline shift. The left-sided hypodensity also appears to have increased June 10, 2024 CT: computed tomography

**Figure 3:**
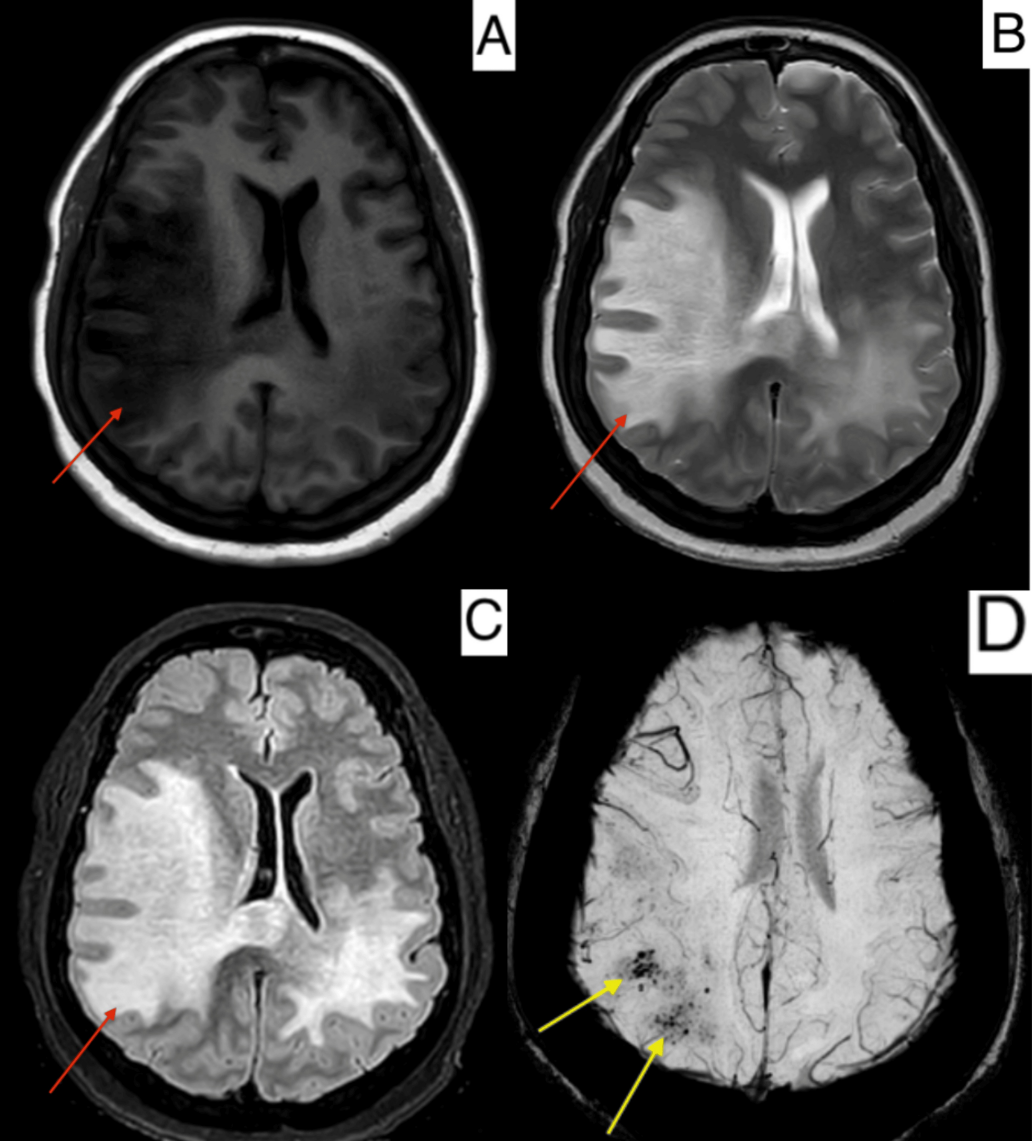
MRI of the brain. T1-weighted image (A), T2-weighted image (B), and T2-FLAIR image (C) showing significant progression of the disease along the splenium of the corpus callosum into the right fronto-parietal-temporal white matter (red arrow). SWI image (D) showing multiple hemorrhagic foci within the right parietal white matter (yellow arrows) June 10, 2024 MRI: magnetic resonance imaging, FLAIR: fluid-attenuated inversion recovery, SWI: susceptibility-weighted imaging

Due to persistent symptoms, the patient was started on IV immunoglobulin (IVIG) therapy [8], a total of 200 grams distributed over five days. On the fifth day, she developed an allergic reaction, itching, and headache, which were resolved with IV pheniramine. So IVIG was withheld. On the same day, June 10, 2024, she became restless and agitated, then progressed to become drowsy, with preferential gaze to the right, new-onset left facial weakness, and left upper and lower limb weakness with a power of 2/5 on both limbs. Emergent CT of the brain showed significant new white matter hypodensity involving the right cerebral hemisphere, causing mass effect and midline shift, features suggestive of fulminant tumefactive demyelination (Figure 2). She was moved to the intensive care unit for close monitoring and started on IV anti-edema measures with hypertonic saline infusions and IV mannitol. She was started back on IVIG therapy along with pulse steroid therapy with IV methylprednisolone. In view of the high viral load of HBV (63,164 IU/mL), she was started on tenofovir, and liver fibrosis was ruled out with ultrasonography [9]. She was electively intubated, and an MRI of her brain was done on the same day, which showed new-onset multiple hemorrhagic foci within the right parietal white matter (Figure 3), features suggestive of AHLE. She was continued on IVIG and pulse steroid therapy. She was extubated the next day and put on supplemental oxygen via a venturi mask.

**Figure 4:**
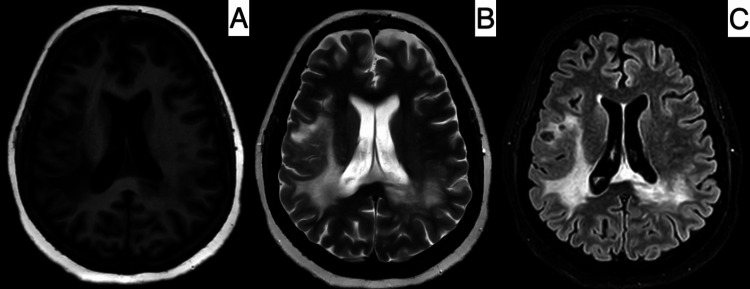
MRI of the brain. T1-weighted image (A), T2-weighted image (B), and T2-FLAIR image (C) showing stabilizing signal changes and reduction in mass effect and midline shift June 25, 2024 MRI: magnetic resonance imaging, FLAIR: fluid-attenuated inversion recovery

She was noted to have made an appreciable improvement in her neurological status. She was continued on IVIG therapy, a total of 200 grams, along with IV methylprednisolone for the next five days. On examination, there was moderate motor power improvement and preserved horizontal gaze movements. She was transferred to the floor, switched to IV dexamethasone, and continued on anti-edema measures, anti-seizure medications, and other supportive medications. A repeat CT brain on June 15, 2024, showed a reduction in midline shift and mass effect. Anti-edema measures were gradually tapered and stopped, and limb physiotherapy was initiated. She was started on oral feeds, which were well tolerated. An MRI of the brain done on June 26, 2024, showed stabilizing signal changes and a further reduction in mass effect and midline shift (Figure 4).

The need for long-term rehabilitation was explained to the patient and family. She was discharged on the same day to a rehabilitation center. At the time of discharge, she was conscious, alert, and oriented, obeyed oral commands, had quadriparesis, with a power of 3/5 in all four limbs, and was hemodynamically stable.

## Discussion

Tumefactive demyelinating lesions (TDL) represent one of the rarest presentations of demyelinating disorders. These lesions are acute, steroid-sensitive, and typically large (>2 cm on T2-weighted MRI) and can mimic tumors due to features such as perilesional edema, mass effect, and broken ring enhancement [10,11]. The pathogenesis of TDLs is believed to be a dysregulated immune response that targets myelin components that coat and protect nerve fibers [10]. This damage can significantly impair the brain's processing centers and communication abilities. The process involves the activation of pro-inflammatory cytokines and immune cells, which in turn causes demyelination, disruption of the blood-brain barrier, and the influx of inflammatory cells into the CNS tissue. This inflammatory sequence results in tissue damage, swelling, neovascularization, and microvascular injury secondary to inflammatory processes [10].

They can occur at any age, manifesting as either a single lesion or multiple lesions [12]. Although primarily associated with MS, TDL may also occur in other conditions such as neuromyelitis optica spectrum disorder, Baló concentric sclerosis, myelinoclastic diffuse sclerosis (Schilder disease), ADEM, AHLE, and autoimmune-mediated encephalitis [10].

In this case, we see the transformation of tumefactive demyelination into AHLE, which is a type of fulminant demyelinating disease causing hemorrhagic white matter lesions [13]. Also known as Hurst disease, it was first described by Hurst et al., who reported two adult patients who developed severe focal neurological signs after respiratory illnesses in 1941 [14]. AHLE causes rapid neurological deterioration that often leads to coma and death; most patients succumb to the disease within six days after symptom onset [14]. The exact cause of AHLE remains unclear, but it is generally considered to be an autoimmune response that develops following a viral or bacterial infection, with most patients experiencing a viral upper respiratory tract infection before diagnosis or less frequently after vaccinations [14]. The pathological mechanism of the disease is the molecular mimicry of viral or bacterial antigens and human myelin antigens that initiate autoimmune demyelination of the CNS neurons [15]. Viral DNA has been detected in brain specimens as well. Brain biopsies of patients with AHLE showed infective agents such as the herpes simplex virus, the varicella-zoster virus, and human herpesvirus-6 [14]. Epstein-Barr virus, hepatitis A, influenza virus, and *Mycoplasma pneumoniae* are also associated with AHLE [1]. There have been no cases of AHLE reported in conjunction with HBV yet.

In this case, the patient demonstrated a high HBV viral load of 63,164 IU/mL; there is a plausible link between her chronic HBV status, the high viral load, and the manifestation of her neurological symptoms. There have been documented cases that suggest a correlation between chronic HBV infection and the onset of demyelinating neuropathies [16], although such occurrences are rare. Chronic HBV has been associated with triggering autoimmune responses, which may lead to various neurological manifestations, including CNS demyelination [16].

The connection between HBV and AHLE can be explained through a series of pathological mechanisms involving immune dysregulation, molecular mimicry, viral neuroinvasion, and a procoagulant state. Chronic HBV infection can lead to persistent immune activation and systemic inflammation, potentially triggering autoimmune processes that may precipitate AHLE [16]. The high HBV viral load in this patient might have exacerbated the immune response, contributing to the severe inflammation seen in AHLE [15]. Additionally, molecular mimicry between HBV proteins and CNS components could lead to cross-reactivity, where the immune system mistakenly attacks the CNS, causing the characteristic demyelination, inflammation, and hemorrhage observed in AHLE. Though HBV is not typically neurotropic, there have been reports of viral antigens in the CNS [17], and in rare cases, the virus might invade the CNS or disrupt the blood-brain barrier, further contributing to the pathology [17].

The diagnosis of AHLE is challenging and often requires a combination of clinical presentation, CSF analysis, and neuroimaging [18]. Brain imaging, particularly MRI scans, shows lesions that tend to be larger than those seen in ADEM, with more edema and mass effect. The lesions often show hemorrhagic foci, which is the distinguishing feature of AHLE [18]. CSF studies typically show pleocytosis with a predominance of polymorphonuclear cells [18]. In some cases, brain biopsies may be performed to examine brain tissue under a microscope, which can reveal the characteristic inflammatory and hemorrhagic changes associated with AHLE [18]. It is important to consider and rule out differential diagnoses with serology such as fulminant MS, viral encephalitis, vasculitis, and venous thrombosis [18].

Treatment of AHLE is aimed at suppressing the autoimmune activity, limiting brain damage, reducing intracranial pressure, and preventing complications. High-dose IV glucocorticosteroids such as methylprednisolone or dexamethasone are often used as pulse steroid therapy for immunosuppression [14]. Additionally, as a second-line treatment, IVIG treatment can help further suppress immune activity and limit white matter damage [13]. Managing increased intracranial pressure, either medically using IV mannitol or hypertonic saline or surgically, is also crucial. Plasmapheresis, a process that involves removing, treating, and returning blood plasma, has also been employed in some cases [19].

Despite a very poor prognosis for AHLE, early diagnosis and aggressive treatment can potentially improve outcomes and save lives, as seen in the case of a 46-year-old woman. Among those who survive, many have significant ailments that warrant long-term rehabilitation aiming to reduce morbidity.

## Conclusions

This case emphasizes the importance of clinicians being vigilant about potential CNS complications in patients with chronic viral infections, particularly those with high viral loads or systemic inflammation. The rapid progression and severity of this condition require a dynamic and multidisciplinary approach to diagnosis. Prompt treatment, as demonstrated in this case, can be crucial for improving outcomes in what is typically a fatal disease. Future directions may include exploring novel therapeutic interventions and establishing standardized protocols to improve outcomes for patients with AHLE.
